# Muslim Communities Learning About Second-hand Smoke in Bangladesh (MCLASS II): study protocol for a cluster randomised controlled trial of a community-based smoke-free homes intervention, with or without Indoor Air Quality feedback

**DOI:** 10.1186/s13063-018-3100-y

**Published:** 2019-01-05

**Authors:** Noreen Mdege, Caroline Fairhurst, Tarana Ferdous, Catherine Hewitt, Rumana Huque, Cath Jackson, Ian Kellar, Steve Parrott, Sean Semple, Aziz Sheikh, Shilpi Swami, Kamran Siddiqi

**Affiliations:** 1 Department of Health Sciences, Faculty of Sciences, University of York, York, YO10 5DD UK; 2 York Trials Unit, Department of Health Sciences, Faculty of Sciences, University of York, York, YO10 5DD UK; 3 ARK Foundation, Suite C-3, C-4, House number 06, Road 109, Dhaka, 1212 Bangladesh; 4 Department of Economics, Dhaka University, Dhaka, Bangladesh; 5 Valid Research Ltd, Sandown House, Sandbeck Way, Wetherby, LS22 7DN UK; 6 School of Psychology, Faculty of Medicine and Health, University of Leeds, Leeds, LS2 9JT UK; 7 Institute for Social Marketing, University of Stirling, Stirling, FK9 4LA UK; 8 Usher Institute of Population Health Sciences and Informatics, University of Edinburgh, Edinburgh, EH8 9AG UK; 9 Hull York Medical School, University of York, Heslington, York, YO10 5DD UK

**Keywords:** Second-hand smoke, Indoor air-quality feedback, Smoke-free homes, Bangladesh, Muslims, Mosque, Imams, Khatibs, Cluster randomised controlled trial

## Abstract

**Background:**

Second-hand smoke (SHS) is a serious health hazard costing 890,000 lives a year globally. Women and children in many economically developing countries are worst affected as smoke-free laws are only partially implemented and homes remain a major source of SHS exposure. There is limited evidence on interventions designed to reduce SHS exposure in homes, especially in community settings. Following a successful pilot, a community-based approach to promote smoke-free homes in Bangladesh, a country with a strong commitment to smoke-free environments but with high levels of SHS exposure, will be evaluated. The study aims to assess the effectiveness and cost-effectiveness of a community-based intervention, Muslims for better Health (M4bH), with or without Indoor Air Quality (IAQ) feedback, in reducing non-smokers’ exposure to SHS in the home.

**Methods/design:**

Based on behaviour-change theories, M4bH and IAQ feedback are designed to discourage people from smoking indoors. M4bH consists of a set of messages couched within mainstream Islamic discourse, delivered weekly by faith leaders (imams and *khatibs*) in mosques over 12 weeks (one message each week). The messages address key determinants of current smoking behaviours including lack of knowledge and misconceptions on specific harms associated with SHS exposure. IAQ feedback consists of personalised information on IAQ measured by a particulate matter (PM_2.5_) monitor within the home. Following adaptation of M4bH and IAQ feedback for the Bangladeshi context, a three-arm cluster randomised controlled trial will be conducted in Dhaka. Forty-five mosques and 1800 households, with at least one smoker and one non-smoker, will be recruited. Mosques will be randomised to: M4bH and IAQ feedback; M4bH alone; or usual services only. The primary outcome is 24-h mean household concentration of indoor fine particulate matter (PM_2.5_) at 12 months post randomisation. Secondary outcomes are 24-h mean household PM_2.5_ at 3 months post randomisation, frequency and severity of respiratory symptoms, health care service use and quality of life. A cost-effectiveness analysis and process evaluation will also be conducted.

**Discussion:**

The MCLASS II trial will test the potential of a community-based intervention to reduce second-hand smoke exposure at home and improve lung health among non-smokers in Bangladesh and beyond.

**Trial registration:**

ISRCTN, ISRCTN49975452. Registered on 11 January 2018.

**Electronic supplementary material:**

The online version of this article (10.1186/s13063-018-3100-y) contains supplementary material, which is available to authorized users.

## Background

Second-hand smoke (SHS) contains 4000 toxic chemicals and is a serious health hazard to non-smokers. Every year, an estimated 890,000 people die and 10·9 million disability-adjusted life years (DALYs) are lost due to SHS exposure, worldwide [1, 2]. A significant proportion of this disease burden (40% deaths and 70% DALYs lost) is due to respiratory conditions, e.g. asthma, chest infections, and lung cancer [3]. Women and children are worst affected: 47% of deaths from SHS exposure occur in female adults and 28% in children [3]. SHS increases children’s risk of acquiring lower respiratory tract infections [4–6], tuberculosis [7, 8], and incident cases, recurrent episodes, and exacerbations of asthma [9]. Parental smoking is also associated with an increased risk of their children’s admissions to hospital [5]. Children living in smoking households are at high risk of becoming adult smokers later [10].

Recognising SHS as a public health threat, comprehensive smoke-free legislation is in place in 55 countries worldwide, including 35 low- and middle-income countries (LMICs), and covers almost 1.5 billion people (20% of the world’s population) [2]. In countries where these bans are comprehensive and strictly enforced, this has led to significantly reduced exposure to SHS and its associated morbidity and mortality [1, 11]. However, compliance to the comprehensive smoke-free legislation is problematic, with only 22 (40%) out of the 55 countries having high compliance rates [2]. In many LMICs smoking bans are only partially implemented. The southeast Asia region, which includes Bangladesh, has the highest burden of disease attributable to SHS in the world. According to the Global Youth Tobacco Survey (GYTS 2007) and Global Adult Tobacco Survey (GTAS 2009), 40% of people living in Bangladesh are exposed to SHS [12, 13]. A recent survey in 12 schools in Dhaka, Bangladesh found that 95% (453/479; 95% CI 92.2 to 96.4) of 9–11-year-old children had saliva cotinine levels consistent with recent exposure to SHS [14]. In total, 43% (208/479) of children lived with at least one smoker, and those living with a smoker had a mean cotinine value approximately double (β = 1.97; 95% CI 1.67 to 2.36) that of those not living with smoker(s) [14]. This indicates that homes remain a key source of SHS exposure in children in Bangladesh.

There is limited high-quality evidence on the effectiveness of interventions that reduce SHS exposure in homes [15], especially in community settings in LMICs. A recent Cochrane review concluded that despite several studies on parental education and counselling programmes, their effectiveness in reducing children’s tobacco smoke exposure has not been clearly demonstrated [15]. Other reviews [16–18] have also highlighted limited evidence and have advocated for better research investigating the effectiveness of such interventions.

This research focusses on community-based approaches to protect non-smoking adults and children from the harms of SHS in their homes. The proposal builds on the findings of a pilot trial conducted in England [19], which concluded that a Smoke-Free Homes (SFH) intervention was acceptable to Muslim communities and feasible to deliver in mosques [20]. It was also possible to recruit, randomise and retain mosques and participant households. There are two other trials (either completed or on-going) in Bangladesh: one evaluating a school-based, smoke-free intervention to encourage children to negotiate smoking restrictions in their households, relying on children as change agents [21]; and another evaluating a multicomponent intervention to reduce home-exposure to SHS during pregnancy in Bangladesh and India (IMPRESS study), relying on pregnant women as change agents [22, 23]. In the present trial, the intervention is directly targeted at smokers (mostly men) working through faith leaders (imams and *khatibs*) and by providing Indoor Air Quality (IAQ) feedback. IAQ feedback has been used successfully with smoking parents of young children in studies in Scotland [24] and in England [25].

## Methods/design

### Aim

This study will evaluate whether a community-based intervention called Muslims for better Health (M4bH) in which imams and *khatibs* will be trained to encourage their congregations in mosques to change their smoking behaviours, with or without IAQ feedback, is effective and cost-effective in reducing SHS exposure in the home.

### Research objectives

The specific research objectives address effectiveness and cost-effectiveness questions (primary objective) as well as implementation questions (secondary objectives).

#### Primary objective

- To investigate the effectiveness and cost-effectiveness of a community-based intervention – M4bH – with or without IAQ feedback, in reducing (i) non-smokers’ exposure to SHS in the home, (ii) frequency and severity of respiratory symptoms, and (iii) health care service use; and in (iv) improving quality of life.

#### Secondary objectives

b. To identify the mechanisms and contextual factors that are likely to influence the impact of M4bH and IAQ feedback
c. To estimate the likely costs and effects of scaling up M4bH with and without IAQ feedback
d. To develop a simple monitoring framework, that could be efficiently employed as the intervention(s) are scaled up, and
e. To identify the likely obstacles to, and opportunities for, implementing and scaling up the intervention(s) and how best to work with communities and policy-makers to overcome the obstacles and maximise the opportunities

### Study design

We will employ an effectiveness-implementation hybrid study design [26] that blends components of effectiveness and implementation research. The study thus consists of three components: (i) effectiveness and cost-effectiveness evaluation (objective a); (ii) process evaluation (objectives b and e); and (iii) implementation and scale-up (objectives c-e ). The study will be conducted over 27 months in total (18 months for (i) and (ii), 6 months for (iii) and another 3 months for analysis and write-up).

**Fig. 1:**
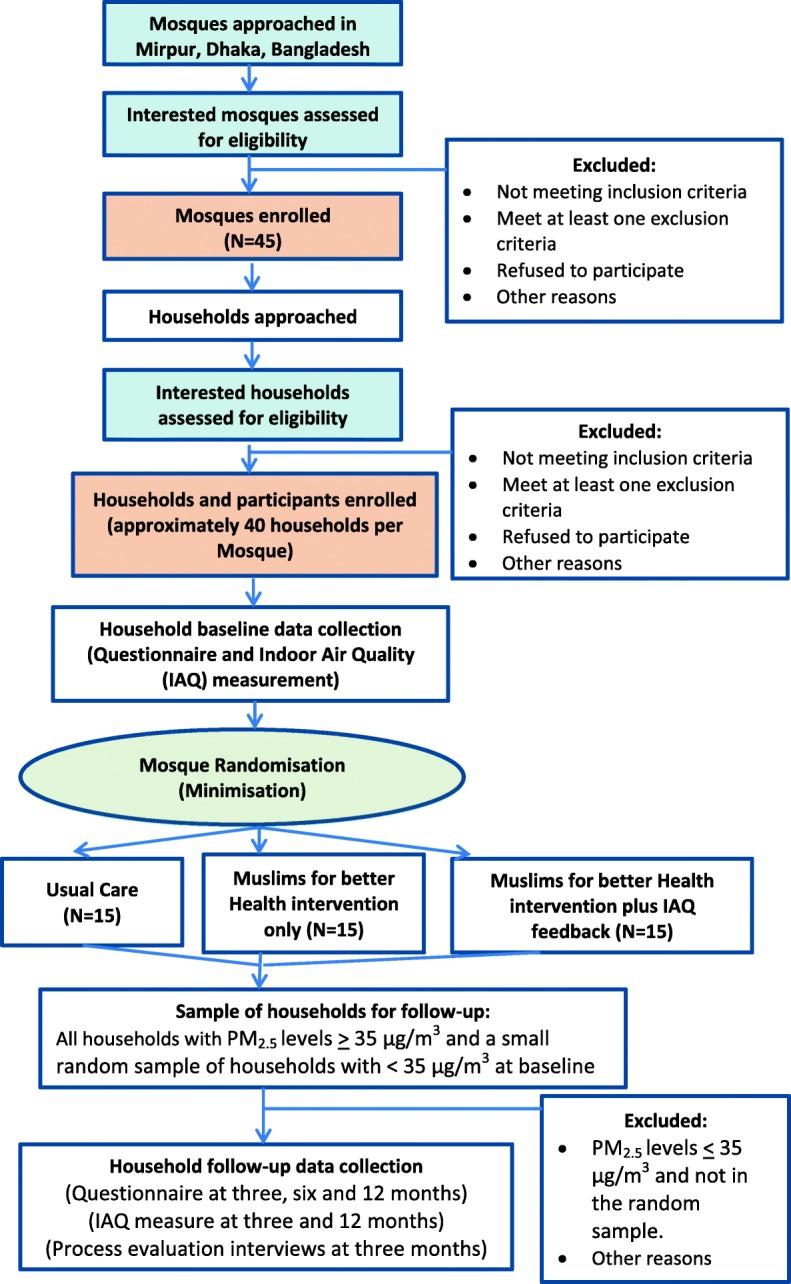
MCLASS II trial flow diagram

Figure 1 shows the design of the trial comprising effectiveness, cost-effectiveness and process evaluation.

### Effectiveness and cost-effectiveness evaluation

A pragmatic, three-arm, open-label, cluster randomised controlled trial (cRCT) with concurrent economic evaluation will be conducted over 18 months in 45 mosques and their catchment communities in Mirpur area of Dhaka, Bangladesh. The three trial arms are as follows:

- Arm 1: M4bH intervention and IAQ feedback
- Arm 2: M4bH intervention alone
- Arm 3: Usual services

#### Intervention description

The interventions have been co-produced with Muslim religious leaders and public health experts in Bangladesh, through an iterative process of adaptation of the SFH intervention developed as part of the MCLASS pilot trial (MR/J000248/1) [19] to the Bangladesh context. IAQ feedback, piloted in Scotland [24] and currently undergoing method development as part of a Medical Research Foundation (MRC) Public Health Intervention Development grant (MR/M026159/1) has also been adapted. These adaptations have been informed by: (1) findings of the qualitative study in the first phase of the IMPRESS study (MR/N006224/1) conducted in Bangladesh and India [23]; and (2) findings from Phase I of the MCLASS II study, which included 30 in-depth interviews with adults in households (a mix of men who smoke in the home/no longer smoke in the home and women whose husbands smoke in the home/no longer smoke in the home) in Dhaka, three focus group discussions with imams and *khatibs* in Dhaka, and five intervention development workshops with representatives of Ministry of Religious Affairs, Islamic Foundation (IF), Imam Training Academy, imams, *khatibs*, muftis and public health experts in Dhaka.

For each cluster (mosque), the intervention period will last for 3 months after randomisation.

##### M4bH intervention

Culturally adapted to a Bangladeshi context, M4bH consists of a set of messages couched within mainstream Islamic discourse, delivered by imams and *khatibs* in mosques over 12 weeks (one message for each week). The messages address key determinants of current smoking behaviours including: lack of knowledge on, and attitudes towards, smoking and SHS exposure by providing information on health consequences of smoking and SHS exposure including addressing any misconceptions; and perceptions about social norms by providing general information on others’ approval. The messages also target the following: prompting intentions; goal setting (both for behaviour, e.g. quit attempt, and the desired outcome of SFH), self-efficacy, commitment, action planning, coping planning, and sources of social support. Each of the messages is supported by at least one verse (*ayah*) from the *Qur’an*, or Islamic faith-based decree including those on addiction, hygiene, health promotion, self-harm and inflicting harm to others, and sanctity of human life (see examples in Table 1 below). The M4bH intervention messages will be delivered to men attending Friday (Juma) prayer congregations in mosques.

**Table 1:** Examples of M4bH intervention messages and supporting ayahs

Ayah	Constructs	Message	Behaviour-change techniques [43]
Surah An-Nisa – 59 (4:59) O you who have believed, obey Allah and obey the Messenger and those in authority among you	Attitude	Wise people like Alims all agree that smoking and indirect smoking are harmful for all. Scientists have also found that there are about 70 types of chemicals in the smoke from second-hand smoking that can cause cancer. Second-hand smoking can also lead to many health problems in newborns and children. Therefore, we have to follow the Prophet’s way to warn ourselves and to warn others, and also listen to wise people	9.1. Credible source 5.1. Information about health consequences 5.2. Salience of consequences 5.6. Information about emotional consequences or 5.3. Information about social and environmental consequences
Surah At-Baqara – 195 (2:195) And do good; indeed, Allah loves the doers of good	Social norms	Those who smoke around us unintentionally harm others directly. Thus, every year 600,000 people die due to exposure to passive smoking worldwide. So, we have to be aware of passive smoking and be careful about smoking inside home and in front of others. We also need to share these messages with others. We must keep ourselves and our families safe from the harm of passive smoking. Allah also loves those who do good things	6.1. Information about others’ approval 5.1. Information about health consequences 5.2. Salience of consequences 5.6. Information about emotional consequences or 5.3. Information about social and environmental consequences
Surah Ar-Ra’d – 11 (13:11) Allah will not change the condition of a people until they change what is in themselves	Self-efficacy (prompt action planning)	You can ask Allah for help to change your situation. But before getting help from Him, we need to take action first. Then, believe that Allah will give you the desired results. It might be difficult for you to stop smoking at home. But will you not do this little thing for the welfare of your family members? How can you then ask God for helping you and your family? So, you just have to take a small step. That is, if you feel the need to smoke whilst at home, go outside the home to smoke	3.1. Social support (unspecified) 1.4. Action planning 1.9. Commitment

Imams and *khatibs* allocated to intervention arms 1 and 2 will receive a half-day training on delivering the M4bH intervention to their congregations using a set of training materials designed specifically for this trial. They will also be provided with a M4bH intervention booklet/guide detailing each message and the supporting verses (*ayahs*) from the *Holy Qur’an* and/or hadith and the order with which the messages are to be delivered over the 12 weeks, to support them in delivering the messages within mainstream Islamic discourse. Imams and *khatibs* in mosques randomised to arms 1 and 2 are also given copies of the M4bH intervention booklet to distribute to members of their congregation after Friday prayers or in study circles, as they feel appropriate.

##### IAQ feedback intervention

**Fig. 2:**
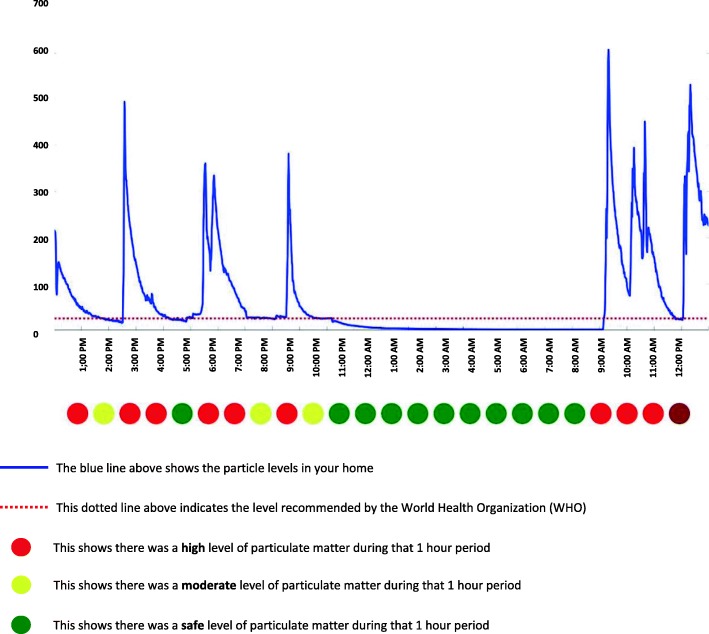
Indoor Air Quality (IAQ) feedback graph

The IAQ feedback intervention will be provided to households in arm 1 of the trial, and will comprise personalised information on the IAQ measured within their home at baseline, in the form of a two-page IAQ feedback leaflet, in order to motivate changes in smoking behaviour in households. The information will be based on data gathered at baseline using a monitor called the Dylos DC1700 (Dylos, Riverside (CA), USA). The first page of the IAQ feedback leaflet will contain information on the total indoor fine particulate matter less than 2.5 μm in diameter (PM_2.5_) concentration measurement time in their home, the mean PM_2.5_ concentration which will be compared to the World Health Organisation (WHO) guidance limit of 25 μg/m^3^ [27], the total time the IAQ was above this guidance limit, and maximum level measured. Based on the Phase-I findings, the feedback leaflet will also have a line graph representing the hourly fluctuations in the indoor PM_2.5_ concentrations within the home with a line representing the WHO guidance limit as a reference, and colour codes summarising levels of particulate matter during 1-h periods (classified as high, moderate or safe) (Fig. 2).

The second page will have pictorial information about the level of 24-h mean PM_2.5_ concentration of that particular home (with classifications hazardous if PM_2.5_ concentration is > 150 μg/m^3^, unhealthy if 36–150 μg/m^3^, moderate if 12–35 μg/m^3^, and good if < 12 μg/m^3^), information about the adverse effects of SHS exposure, recommendations to reduce SHS exposure in the home, and a target that is achievable by implementing SFH rules within that home. This will be accessible to individuals with a diverse range of literacy and numeracy skills. Trial field investigators (FIs) will deliver and discuss the IAQ feedback with members of the households in person and answer any question raised, which may take approximately 10 min.

Follow-up IAQ measurements will take place at 3 months and 12 months in 30 homes per mosque where high levels of SHS (≥ 35 μg/m^3^) were identified at baseline, plus a small number of households under this threshold if necessary. All followed up homes in all trial arms will receive details of their month-12 IAQ measurements and feedback after trial completion.

##### Usual service

No intervention will be offered to mosques randomised to the usual service arm until the trial has completed. Following the completion of the pilot trial, mosques will be offered the M4bH toolkit free of charge.

#### Recruitment

We aim to enrol 45 mosques (15 in each arm) and 1800 households (40 from each mosque catchment area) into the trial.

##### Mosque eligibility criteria

A mosque will be eligible if it:

- Is based in the residential parts of Mirpur, Dhaka
- Hosts communal prayers (including Friday prayers)
- Is at least half a kilometre from another participating mosque
- Has an imam or *khatib* who is a self-reported non-smoker
- Is enlisted with the Islamic Foundation (IF). These mosques will be under a government ministry and will be monitored by the government

A mosque will not be eligible if it:

- Is located in an area with restricted access such that it is difficult to get in and recruit households
- Has too small a catchment area to recruit at least 40 households from
- Does not have an imam or *khatib* who is willing to participate and deliver the M4bH intervention

##### Household eligibility criteria

For this trial, a household is defined as a single housing unit shared by one or more individuals.

For a household to be eligible for the trial, it should:

- Have at least one resident attending one of the participating mosques
- Have at least one adult resident who smokes cigarettes or other forms of smoked tobacco (e.g. bidi, waterpipe) regularly (at least 25 out of 30 days/month)
- Have at least one non-smoking resident of any age
- Not be planning to move home in the next 12 months

In circumstances where two or more families share a housing unit and stay together they will be enrolled as one household if all family heads agree to participate. The families will have to agree who would be the household lead for the trial.

A resident is defined as an adult or child who has been staying in the home for at least the previous 3 months and plans to stay for at least one more year in the home.

A household will not be eligible if:

- It uses coal and/or biomass fuel for domestic use
- The household head/lead is unwilling/unable to give written informed consent
- One or more families in a shared housing unit do not want/do not agree to participate

##### Mosque recruitment

FIs will collect Global Positioning System coordinates for mosques that are IF enlisted. Those that are more than half a kilometre away from another participating mosque will be approached via chairs of their respective committees and other relevant leaders (e.g. ward commissioners) to seek their expression of interest in participating in the MCLASS II trial. Existing links with the IF, including its local community officers, as well as links with mosque committee members will be used in making the initial contact. All interested mosques will be visited and their committee chairs and leaders met to inform them about the trial including explaining random allocation and its purpose. If interested in trial participation, mosques will be screened for eligibility and provided with an information sheet including: the aims and objectives of the trial; a description of the trial interventions and trial arms; details about the randomisation process; the role of the mosque and imams/*khatibs* in the trial; withdrawal processes; risks, advantages and disadvantages of participating; how the results will be used; and confidentiality issues.

##### Mosque agreement to participate

Agreement to participate in the trial will be sought from the mosque imam/*khatib* by FIs. They will be asked to sign a written agreement to participate for their mosque and themselves. This approach is considered to be reasonable given the organisational structures within mosques based on the MCLASS pilot trial findings [19].

Agreement to participate will be sought for:

- Implementing the M4bH intervention in the mosque, should that mosque be allocated to one of the M4bH groups
- Facilitating the research team (recruitment officers) in the recruitment of participants in the respective mosque catchment area
- Approaching mosque imams and *khatibs* to seek their consent to take part in interviews, and
- Recording of non-identifiable mosque data according to the trial protocol

##### Household recruitment

Local researchers will approach Muslim community household heads, generally men, living in the catchment area and attending prayers in the participating mosques. Local researchers will be supported in the field by supervisors and caretakers of the IF who work in the area and are known to the community.

A number of strategies will be used to recruit participants:

1. We will provide imams, *khatibs* or any other relevant persons from the mosque with a written script that they will use in announcements to inform members of their congregation about the study
2. At the end of Friday sermons (just before the Friday prayer), imams/*khatibs* will introduce an MCLASS II researcher who will introduce the trial to the audience in less than 5 min
3. The FIs, with the support of the imams, will also visit households in the catchment areas and provide households with information about the study

##### Household eligibility assessment

Each potential household will be allocated a unique screening number, which will be used as their unique trial identifier (ID) if enrolled in the trial. The participating household members will also receive trial IDs that will be unique for each participant but will include the household number so as to be able to identify participants from the same household. The following anonymised household screening information will be collected: mosque attended and eligibility for inclusion using the eligibility criteria described above.

The following will be recorded for those eligible:

- Consented
- Declined
- Reason for declining, if given

For those not eligible for the trial, the reason for exclusion will be recorded.

##### Informed consent for households and participants

Since the M4bH intervention is to be delivered in the mosque, agreement for delivering the intervention will be sought from the mosque imams and *khatibs* and not from individual participants. This approach is proportionate given the very low risk associated with participation and the likely potential benefits of the intervention. However, written informed consent will be sought from all adult participants in the household for all other research activities.

Based on the main eligibility criteria, i.e. households with at least one adult resident who smokes cigarettes or other form(s) of smoked tobacco (e.g. bidi, waterpipe) and at least another non-smoking resident of any age, eligible households will fall into two possible categories:

- Households with at least one adult smoker and one child: In this case, consent will be sought from all the adult residents for completing the baseline (and follow-up when requested) questionnaire. Consent will also be sought from parents/guardians of any child aged below 18 years old, to collect baseline (and follow-up when requested) data on the child
- Households with adult residents only including at least one smoking and one non-smoking adult: consent will be sought from all adult residents for completing the baseline (and follow-up when requested) questionnaire

Consent will also be sought from the household head/lead for the participation of the household, including IAQ measurement which will involve installing the Dylos DC 1700 for at least 24 h in their homes before and at 3 and 12 months after randomisation. Installation will involve plugging the meter into the electricity mains in the living area of the house (excluding the kitchen) most commonly used by family members.

FIs will go through the respective information sheet with potential participants during their appointment and seek consent(s) as appropriate. Participants are not offered any personal incentive for taking part in the trial; however, each household will receive Taka 200 (GBP 2) at baseline and the 12-month follow-up time point, to compensate for the time they are giving, and to cover expenses associated with powering the air-quality monitors in their home for 24 h on up to three occasions.

Informed consent will be obtained prior to registration of participants and before any trial-specific baseline assessments.

##### Household and participant registration

For all individuals who have expressed an interest and are, therefore, approached for potential inclusion, the following non-identifiable data will be recorded in secure trial databases: mosque attended; date of birth; gender; smoking status; eligibility criteria; consent given/refused; and reasons for not consenting (if given). The time taken for screening will also be recorded.

All consenting individuals (and households) will be registered in a secure trial database, using their name, date of birth, address, and unique trial ID (both for households and individuals). Only the chief investigator, trial coordinator, and researchers involved in collecting, quality checking and entering data will have access to these identifiable data at any stage of the trial. For the purpose of baseline and follow-up data collection and conducting analyses, only the unique trial ID will be used, thereby ensuring anonymity of data.

Paper consent and agreement to participate forms will be kept in a locked filing cabinet at the ARK Foundation in Dhaka, separate from the rest of the trial data.

#### Baseline assessments

##### Mosque baseline assessment

For each mosque participating in the trial the following information will be recorded at the start of the trial:

- Catchment area
- Type of mosque (ethnic and religious denomination)
- Average estimate of number of people who attend two or more daily prayers
- Average estimated size of Friday congregation
- Average estimated size of study circle (men)
- Average estimated size of *Qur’an* class
- Average age (self-reported by teacher) of students/children taught
- Average estimated size of study circle (women)

A semi-structured questionnaire will be utilised to measure the pre-intervention training knowledge of the imams/*khatibs* on smoking and SHS exposure.

##### Participant (and household) baseline assessment

Baseline data will be collected from households, and all consenting participants in the household, once informed consent has been provided and before randomisation of their mosque, using questionnaires.

The following data will be collected about the household:

- Presence of outside space; number of bedrooms; number of residents (adults and children); number of resident smokers (adults and children)
- Self-reported smoking restrictions, or lack thereof, in the home
- Number of shops that sell tobacco products in neighbourhood
- Presence of household amenities (e.g. electricity, flush toilet, etc.)
- Type of fuel used for cooking
- Presence of mould/moisture in homes and any damage caused
- Presence of cattle/pets/poultry in home
- Mosque attendance

IAQ of the household will also be measured at baseline as 24-h mean levels of PM_2.5_ concentration using the Dylos DC 1700 meter.

The following data will be collected about all consenting participants in the household:

- Socio-demographic variables (date of birth, gender, education)
- Self-reported smoking behaviour (≥ 11 years only)
- St George’s Respiratory Questionnaire (SGRQ) ≥ 11 years; frequency and severity of respiratory symptoms for under 11 year-olds
- Health service use
- Health-related quality of life: (EQ-5D-5 L for 18 years and over, Proxy version of the EQ-5D-Y: 1 for 11–17 year olds, PedsQL version 4.0 for under 11 year-olds)
- Attitudes, social norms, intentions and action planning, self-efficacy, and coping planning with regards to smoking and SHS exposure (≥ 18 years only)

#### Randomisation process

Since the M4bH intervention is an ‘educational’ intervention delivered at a group level, this trial is a cRCT where mosques will be randomly allocated to one of the three trial arms.

Mosque, household and participant recruitment, baseline data collection and randomisation of the 45 participating mosques will be conducted over a period of 6 months. Once recruitment has ceased and mosque and household baseline data collection are complete within a particular mosque, the mosque will be randomly allocated 1:1:1 to one of the three arms using minimisation to ensure balance across the groups on the average estimate size of the Friday prayer congregation (≤ 1500/> 1500) and geographical location (wards within the Mirpur area of Dhaka). Random allocation will be performed by a statistician at the University of York not involved in the recruitment of mosques or households, thus ensuring allocation concealment.

#### Contamination and risk of bias

The process of randomisation will minimise the chance of selection bias. There is a possibility that mosques/or households in the control arms could be exposed to the M4bH intervention. All mosques in the trial area will be identified, including a list of mosques enlisted with the IF. A geographical map of these potential mosques will be prepared and Geographic Information System maps used to ensure that the catchment areas of any two clusters do not overlap. A buffer zone of half a kilometre between mosques will be used to minimise the risk of contamination.

By the nature of the interventions used within this trial, blinding of the participants and the imams and *khatibs* delivering the intervention is not possible. Outcome data collection and data analysis are also not blinded.

In order to minimise loss to follow-up, the household head/lead will be requested to inform the trial team of any relevant changes in the household. FIs will also be in regular contact with the household head/lead over the phone. Households will be informed about follow-up prior to the date. Information materials in the appropriate language will be provided to ensure that participants understand clearly what the expectations would be if they decide and give consent to participate in the trial.

#### Withdrawal

Households and participants will be free to withdraw consent and leave the trial at any time without giving a reason. Written information on who to contact if they wish to withdraw will be provided to all participants. They will be able to withdraw by letting any member of the research team know if they wish to do so. If a household or participant withdraws consent to participate, no further data will be collected from them. However, data collected up to the point of withdrawal will be retained and used in the analysis, except where withdrawal of consent for the use of this data is explicit, in which case all data will be destroyed.

#### Sample size

Baseline air particulate data were considered for the households recruited from the first six MCLASS II study mosques to be randomised into the trial. Of the 240 recruited, 222 households had at least 22 h of PM_2.5_ concentration measurement at baseline, with an average PM_2.5_ of 66.5 μg/m^3^. The United States (US) Environmental Protection Agency (EPS) considers an Air Quality Index (AQI) of 101 (equivalent to 35 μg/m^3^) or more to indicate unhealthy levels of air pollutants for sensitive groups such as children and the elderly (https://airnow.gov/index.cfm?action=aqibasics.aqi) [28]. Eighty percent of the 222 households at baseline had an average 24 h PM_2.5_ of 35 or more; among these households the mean (standard deviation; SD) was 75.9 (44.2) μg/m^3^.

We propose to recruit 45 clusters (mosques) and 40 households with at least one resident smoker per cluster, and shall follow up up to 30 households per mosque with average baseline PM_2.5_ of 35 μg/m^3^ or more. If there are more than 30 households in the mosque with average baseline PM_2.5_ of 35 μg/m^3^ or more, then 30 will be randomly selected for PM_2.5_ follow-up at 3 months, with randomly selected reserves for those households that are lost to follow-up at this time. If there are less than 30 households in the mosque with average baseline PM_2.5_ of 35 μg/m^3^ or more, then the shortfall will be made up of randomly selected households with PM_2.5_ under 35 μg/m^3^. The same households followed up at 3 months will be contacted for follow-up again at 12 months. We shall assume a 20% attrition rate at 12 months (the primary time point).

The intracluster correlation coefficient (ICC) for salivary cotinine level was negligible in the MCLASS trial (< 0.01) [19], but we shall assume an ICC of 0.02 in this trial to be conservative. With these figures, we would have 90% power to detect an effect size of 0.3; this is equivalent to a difference of 13.5 (e.g. from 76 μg/m^3^ to 62.5 μg/m^3^) assuming a SD of 45, between each intervention group and the control group, using a two-sided 5% significance level. In practice, we would expect to have greater than 90% power with this sample size by virtue of adjusting the analysis for baseline PM_2.5_ concentrations, which we would expect to be predictive of the follow-up measurement. We have not accounted for this potential pre-post correlation to ensure that the calculation is conservative, and to minimise the risk of the trial being underpowered. In the trial analysis, we may wish to compare the two intervention arms. The difference between these two arms is likely to be much smaller than one we could expect to observe between one of the intervention arms and the control arm. With these figures, we will retain 80% power to detect a smaller effect size of 0.2 between the two intervention groups, assuming a pre-post correlation of 0.6.

#### Outcomes

Post-randomisation outcome data will be collected from households: (1) where 24-h mean PM_2.5_ levels are 35 μg/m^3^ or above (we envisage approximately 75% (*n* = 30) households per cluster) and (2) in a small random sample of households where 24-h mean PM_2.5_ levels are below 35 μg/m^3^, as described in the ‘Sample size’ section.

##### Primary outcome

The primary outcome will be 24-h mean household indoor SHS concentration measured as fine particulate matter less than 2.5 μm diameter (PM_2.5_) at 12 months post randomisation.

PM_2.5_ will be measured in homes using the Dylos DC 1700 (Dylos, Riverside, CA, USA) a low-cost particulate counter validated for use in domestic settings [29]. Data from smokers’ homes in Scotland suggest that there is little difference between PM_2.5_ levels measured on the first day compared with levels measured over the following period of up to 6 days. This suggests that installation of these monitors for 24 h will provide a good representation of SHS levels within that home.

*Setting up the Dylos*: 24-h mean PM_2.5_ concentrations will be calculated for each household before randomisation, and at 3 and 12 months post randomisation. Within each household, the Dylos will be plugged into the electricity mains in the living area of the house (excluding the kitchen) most commonly used by family members, at least 1 m away from any doors, windows, or obvious potential sources of PM_2.5_. The Dylos will be switched on to start the logging process at the beginning of each data collection period and will be left to measure and log 1-min particle number concentrations for the duration. The monitors will be supplied with 6-h backup batteries in case of power cuts or brown-outs. Devices record IAQ every minute and enable estimation of SHS concentrations with 1-min resolution over the sampling period. The team is aware of the sensitivities of measuring IAQ in homes and has experience of doing so in various LMICs including Mexico, Pakistan, Indonesia, Chad, Bangladesh, and India [30]. The team will work closely with local partners to ensure that gender and cultural practices are respected.

*Analysing data from Dylos*: data from the Dylos machine will be downloaded to a desktop portable computer at the ARK foundation in Dhaka using the AFRESH software version 4.2 at the end of each sampling day. Dylos particle number concentrations will be converted to equivalent PM_2.5_ mass concentrations and corrected for non-linearity of response. Entry and exit times for each venue will be matched to the sampling day record sheets and an average PM_2.5_ concentration will be calculated for each household. Feedback graphs (Fig. 2) presenting baseline measurement data will be generated to be used in the IAQ feedback component for arm 1.

##### Secondary outcomes

The following will be measured for the household:

- SHS concentration measured as 24-h mean PM_2.5_ concentration at 3 months post randomisation
- Smoking restrictions at home: the level of smoking restrictions at home will be assessed through a questionnaire directed at the adults in the households at 3, 6 and 12 months post randomisation

The following will be measured for each member of the household:

- Frequency and severity of respiratory symptoms: for participants aged 11 years and over, Part 1 (eight questions) of the validated SGRQ [31] will be used to assess participants’ recollection of their respiratory symptoms over the preceding month at 3, 6, and 12 months postrandomisation. SGRQ is a validated questionnaire and a Bangla translation is available for use. For participants younger than 11, respiratory symptoms will be assessed by another severity scale developed and validated by Chauhan et al. [32] at 3, 6, and 12 months post randomisation
- Quality of life: at 3, 6, and 12 months post randomisation the EQ-5D [33, 34] will be used for adults 18 years and over, EQ-5D-Y [35, 36] for adolescents (11–17 years inclusive) and PedsQoL [37] for children aged below 11 years to measure quality of life
- Health services use: a health service utilisation questionnaire previously used in the MCLASS pilot trial [19], and adapted to the Bangladesh context, will be used to collect number and type of contacts with physicians, hospital admissions, pharmacy visits and medication prescriptions for all participants. This will be assessed at 3, 6, and 12 months’ follow-up. The MCLASS pilot identified questionnaire items with very low frequency of responses and these questions are omitted from the MCLASS II service use questionnaires
- Mediators of intervention effectiveness: mediators of intervention effectiveness will be quantitatively investigated focussing on those constructs that map onto the intervention logic model that are: attitude, social norms, intentions and action planning, self-efficacy and coping planning with regards to smoking and SHS exposure. These will be assessed at baseline, and at 3, 6, and 12 months’ follow-up using a pre-tested questionnaire, from adults (aged 18 years and over only)

In addition to socio-demographic variables, other confounders identified through the literature a priori will also be measured including:

- Number of residents
- Building environment
- Neighbourhood
- Presence of mould/moisture in homes
- Use of gas for cooking or gas/kerosene/oil heater.
- Mosque attendance and receipt/participation in M4bH programmes

The following will be measured for the imams and *khatibs* who will deliver the trial intervention:

- Pre- and post-intervention training knowledge on smoking and SHS exposure using a semi-structured questionnaire

#### Frequency and duration of follow-up

**Fig. 3:**
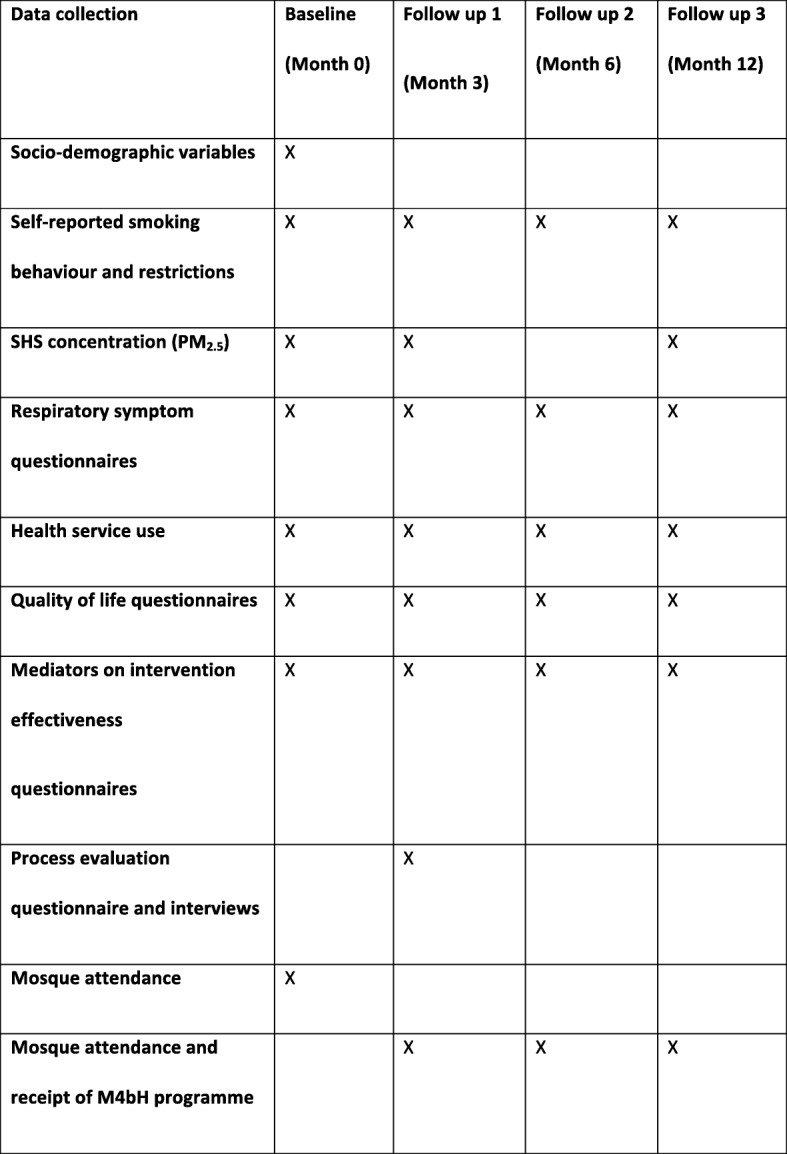
Data collection schedule

Data will be collected at baseline, and 3, 6, and 12 months post randomisation as indicated in Fig. 3 below.

#### Data collection

A total of 16 FIs will screen, recruit households, and collect baseline and follow-up information from 1800 households in 18 months. The FIs will receive 3 days’ training on trial procedures including taking informed consent, administering and completing the questionnaires, delivering IAQ feedback, and ethical issues such as autonomy of households and individual participants on making decisions about participation, freedom to withdraw from the trial without giving any reason or consequence, privacy, confidentiality, anonymity. They will work in pairs during recruitment and baseline data collection for the first few mosques (approximately six mosques) and then work individually with each FI working with one mosque at a time.

Data will be collected using paper-based questionnaires designed specifically for the trial. Collected data will be quality checked and entered into the secure, password-protected database designed specifically for this trial on REDCap (Research Electronic Data Capture), a secure web application for building and managing online surveys and databases (https://projectredcap.org/software/). Collected data will be stored on a central database server.

All data will be stored and transferred following Health Insurance Portability and Accountability Act protocol. The staff involved in the trial will be trained on data protection processes. The staff will be strictly monitored to ensure compliance with privacy standards.

#### Statistical analysis of effectiveness data

##### Analysis of clinical data

The trial will be analysed and reported according to the Consolidated Standards of Reporting Trials (CONSORT) guidelines extension for cluster trials [38]. A detailed statistical analysis plan has been prepared and reviewed by an independent Trial Steering Committee (TSC) prior to the completion of outcome data collection. All analyses will be conducted following the principles of intention-to-treat (ITT), including all participating households within clusters in the trial arm to which the cluster was randomised, using two-sided statistical tests at the 5% significance level. Summaries of the baseline characteristics of the clusters, households and participants will be presented by trial arm. Continuous measures will be reported using descriptive statistics (e.g. *n*, mean, SD, median, minimum and maximum) and categorical data as counts and percentages. No formal statistical comparisons by trial arm will be undertaken on baseline data.

Screening recruitment, and retention data for mosques and households will be summarised, and a CONSORT flow diagram produced. Reasons for non-eligibility/participation will be reported, where available. Follow-up and withdrawal rates at each time point will be presented by randomised group, with reasons for withdrawals given where available.

All outcome data will be summarised descriptively by randomised group and time point.

##### Primary analysis

Twenty-four-hour mean household PM_2.5_ at 3 and 12 months post randomisation will be compared between the groups using a linear covariance-pattern mixed model incorporating the two post randomisation time points and controlling for pertinent baseline covariates at the household and cluster level (including baseline PM_2.5_ value (household-level) and the factors used in the minimisation (cluster-level)). Clustering at the mosque level will be accounted for using a random effect. The correlation of observations within households over time will be modelled by a covariance structure. The mean difference at 12 months will serve as the primary outcome, and the difference at 3 months as a secondary outcome. Residuals will be checked for normality and transformations for the PM_2.5_ data (e.g. log) will be considered. Parameter estimates and corresponding 95% confidence intervals will be presented.

We hypothesise that a combination of the M4bH intervention and IAQ feedback is more effective in improving IAQ than usual services. Therefore, the comparison between the M4bH intervention plus IAQ feedback (arm 1) and usual services (arm 3) will serve as the primary comparison, whilst the comparison between M4bH intervention alone (arm 2) and usual services (arm 3), and between M4bH intervention plus IAQ feedback (arm 1) and M4bH intervention alone (arm 2), will serve as secondary investigations.

##### Sensitivity analyses

To account for non-compliance with the intervention, a complier average causal effect (CACE) analysis [39] will be considered which provides an unbiased estimate of the treatment effect in the event of non-compliance.

##### Secondary analyses

Respiratory symptom questionnaire scores from months 3, 6, and 12 will be analysed in an analogous way as PM_2.5_ concentrations, with an additional random effect for household if feasible. Self-reported smoking behaviour and restrictions will be summarised descriptively per arm.

To explore the potential for mediating mechanisms, the primary analysis model will be repeated, but this time including the mediator of interest as the outcome. The primary analysis will then be repeated including the mediator as a fixed effect. We will be looking for the intervention effect being reduced and the mediator effect being large. Further details including a full list of potential mediators to be explored will be pre-specified and detailed in the statistical analysis plan. These analyses will be exploratory and interpreted accordingly.

#### Analysis of economic and quality-of-life data

An incremental cost-effectiveness analysis will be conducted to estimate the value for money afforded by the M4bH intervention with and without IAQ feedback over and above usual care.

##### Intervention costs

The costs of providing M4bH with and without IAQ feedback will be calculated. Costs will include the staff time required to deliver the M4bH intervention and the cost of materials used. Additional costs in the IAQ feedback arm include the costs of the Dylos DC1700 and the time taken to provide feedback to the household.

##### Training costs

The costs of training of individuals to deliver the intervention will be calculated. Training requires staff time of the trainer plus staff travel cost. Staff time is based on the salary of the trainer and allocated on a cost per minute basis plus costs of materials. Training costs are divided by the number of trained adults at each site. Training cost per adult benefits from economies of scale whereby cost per adult decreases as the number of adults trained increases at a site.

##### Health care utilisation

Health care resource use data for each participating member of the household will be collected in all three trial arms at baseline and 3, 6, and 12 months’ follow-up. This data will allow the calculation of cost profiles for each individual based on local unit costs of care which are multiplied by quantities of resources consumed to calculate a per individual cost.

##### Quality-adjusted life years

EQ-5D [33, 34] will be used for adults aged 18 years and over, EQ-5D-Y [35, 36] for adolescents (11–17 years) and PedsQoL (version 4) [37] for children aged younger than 11 years at baseline and 3, 6, and 12 months’ follow-up to calculate changes in Quality-adjusted Life Years (QALYs) for all household members using local social tariff scores to derive health utilities. QALYs will be calculated by using the area under the curve between baseline and follow-up assuming a linear change between recorded points [40].

#### Assessment of cost-effectiveness

##### Base case analysis

The outcome for the cost-effectiveness analysis will be QALYs at 12 months and the cost-effectiveness analysis will be performed at this time point. The costs will include intervention cost and the cost of health resources during the 12-month period post randomisation. QALYs will be calculated during the same time period. An incremental cost-effectiveness analysis will be performed to estimate the incremental cost-effectiveness ratios (ICERs). Both costs and QALYs will be combined to calculate the incremental cost per QALY. ICERs will be calculated for M4bH and IAQ feedback over and above usual care, M4bH intervention only over and above usual care, and M4bH plus IAQ compared to M4bH alone.

##### Uncertainty assessment

Sensitivity analysis is conducted by varying the cost components of the intervention to estimate the robustness of the cost-effectiveness ratios. Uncertainty around the decision to adopt the intervention will be assessed through a non-parametric bootstrap re-sampling technique. Bootstrapping has been proposed as an efficient approach for calculating the confidence limits for the ICER as its validity does not require any specific assumptions with regard to the underlying distribution. A cost-effectiveness acceptability curve (CEAC) will be plotted based on the outcomes of the 5000 bootstrap replications [41].

#### Interim analyses

No interim analyses will be conducted.

### Process evaluation

The process evaluation will be carried out concurrently with the effectiveness and economic evaluations. The three key functions of a process evaluation for an effectiveness trial, identified by the MRC guidance for process evaluation [42] – mechanisms of impact, context, and implementation (including intervention fidelity) will be explored as secondary outcomes.

#### Mechanisms of impact

To capture the views and experiences of participants, all household head/lead participants in both intervention arms whose homes have been included in the follow-up sample will complete a short questionnaire at the end of the intervention, as part of month-3 follow-up, exploring which components of the M4bH intervention and IAQ feedback they engaged with, the acceptability of each component and any perceived benefits/non-benefits to themselves and their families including those which were unanticipated. A purposive sample of 15–20 participants, a mix of men and women from households who have/have not achieved SFH (based on PM_2.5_ scores) will be interviewed to explore key issues that emerged in the questionnaire; for example, any messages worded within the mainstream Islamic discourse that were seen to be particularly influential or inappropriate. Based on the team’s previous experience, to avoid participant fatigue with data collection, these interviews will be administered a few days after the questionnaire.

#### Context and implementation

In six purposively selected mosques (a mix of those scoring high and low on fidelity) the imams and *khatibs* who delivered the intervention will be interviewed to explore how contextual factors, such as the mosque environment and other social, economic, cultural, environmental and political factors, have influenced the delivery and impact of the interventions. These in-depth interviews will also explore implementation issues including perceptions of the potential reach of M4bH, likely obstacles and potential opportunities for scale-up of M4bH.

#### Intervention fidelity assessment

A fidelity index, mapped onto the behaviour-change techniques [43] that underpin the M4bH intervention, will be used to assess adherence to delivering the intervention. Each mosque will deliver the intervention for a maximum of 12 weeks. It is expected that there will be at least 360 intervention sessions over this period (one session per week × 12 weeks per mosque × 30 mosques). The researcher(s) will perform the fidelity check on 10% of the sessions (approximately 36 observation sessions in total) by observing 50% of sessions in six randomly selected mosques in the intervention arms (three mosques per each intervention arm). The researcher(s) will code the behavioural change techniques applied during the sermon using a standardised coding fidelity index.

#### Process evaluation data analysis

##### Quantitative data analysis

The quantitative data from the short process evaluation questionnaires will be analysed using descriptive statistics.

##### Qualitative data analysis

The qualitative data from the short process evaluation questionnaires will be analysed using content analysis [44]. Interviews with imams and *khatibs* will be transcribed verbatim and translated into English and analysed using the Framework approach which is designed to address applied programme and policy-related questions [45]. NVivo 11 software will aid data handling. Integration of interview findings with respective short questionnaire data will be done using a ‘triangulation protocol’ [46].

##### Intervention fidelity data analyses

The intervention fidelity data will be analysed descriptively.

### Implementation and scale-up

Based on a recent review [47] of the success factors for scaling up public health interventions, the implementation and scale-up phase will involve three activities:

#### Budget impact analysis

A budget impact analysis will be conducted using secondary data to calculate the number of households who might benefit from the implementation of the M4bH intervention, both with and without feedback, and the associated cost of monitors. Based on estimates of the numbers of households, the budget impact of providing the M4bH intervention to all households who might benefit, both with and without a monitor, will be estimated. This component is important as it will demonstrate the potential cost which would be taken into account when assessing the affordability of intervention rollout.

#### Monitoring framework development

A simple monitoring framework which could be efficiently employed as the intervention gets disseminated widely will be developed. An expert panel will first examine the evaluative framework used in the effect and economic evaluation within MCLASS II and develop a consensus on a set of measure that could replace it during scale-up of M4bH.

#### ‘Way Forward’ workshop

The findings of the two activities above, as well as the imam and *khatib i*nterviews during process evaluation, will be presented to policy-makers, development partners, and respective faith-based and civil society organisations in a final ‘Way Forward’ workshop. The finalised intervention resources will also be presented. Participants will be facilitated to consider ways to overcome the identified blocks to implementation and to plan how to optimise opportunities for effective scale-up.

## Discussion

The MCLASS II study focusses on reducing SHS exposure in homes as a means of reducing the burden of lung diseases in LMICs. There is a need for measures to protect non-smokers, particularly women and children from SHS exposure within the home. This study addresses a major evidence gap in this area, which is partly responsible for no clear guidance on how to implement smoking restrictions and protect non-smokers from SHS exposure in homes.

The study evaluates an innovative intervention comprising messages to promote SFH embedded within the Islamic discourse and delivered by faith leaders – imams and *khatibs –*to their congregations, with or without IAQ feedback. If found to be effective in changing smoking behaviour in homes, such an approach could shift the existing smoking norms, i.e. reducing visibility of smoking in social spaces and de-normalising it for children who might otherwise take up smoking. Previous studies in South Asia (one in Pakistan and another in Bangladesh) showed promise in community-based approaches in reducing visibility of smoking in social spaces and promoting SFH [48, 49]. In addition, research findings from this study are likely to be generalisable, particularly to those communities that are facing high tobacco-related disease burden with similar smoking norms, and places a high value on faith-based settings and leaders in their public and private lives. The intervention also lends itself for adaptation and to be used in influencing other unhealthy behaviours through faith-based settings resulting in improving family health in ways that goes beyond to what is proposed here.

With IAQ measurements for 1800 households at baseline, the study will provide a large dataset on the magnitude of SHS exposure within the home, as well as any key issues around IAQ measurement, in Bangladesh. IAQ feedback has a potential to bring about change in smoking behaviour in or around the home for some individuals [50], thus can complement other community-based interventions such as M4bH.

The effectiveness-implementation hybrid design utilised in this study has a distinct advantage of allowing for the gathering of data on the delivery of an intervention during an effectiveness trial that inform its potential for implementation and scaling up in the ‘real world’. MCLASS II will be conducted with active engagement of policy-makers, a range of implementers and target communities. The intervention and its implementation will be tailored to the local context, which will be informed by qualitative research within target communities. A strong political will exists among policy-makers who are fully aware of the disease burden associated with SHS, the relevant gap in policy and its implementation, and the potential benefit of addressing SHS exposure in homes. Their engagement and interest in this and the other two MRC-funded studies on SFH in Bangladesh is evidence of the priority given to this area. Costs will be measured, economic modelling of scaling up SFH conducted, and an efficient monitoring framework developed. These are key success factors for implementation and scale-up [47]. Moreover, to enhance the impact of the research, a robust dissemination strategy will be offered consisting of (1) engagement with, and contributing to, policy groups through existing membership of policy advisory boards and working groups (WHO policy advisory groups, and The International Union against Lung Disease and Tuberculosis policy sub-groups); (2) working with the IF and the Ministry of Religious affairs to ensure incorporation of the M4bH training package within imams and *khatibs* training curricula; and (3) producing effective dissemination materials, i.e. policy briefs, publications in high-impact journals, presentations and seminars at conferences, press releases, media reports, web-publishing, and social media feeds. It is anticipated that the results of this study will be published in 2020.

All these elements of the study have the potential to influence policy and practice on tobacco control, particularly SHS exposure, in Bangladesh (Additional file 1).

## Trial status

MCLASS II Protocol version 4.0, 29 May 2018. The MCLASS II trial began on 11 April 2018 with an expected end date of July 2020. We are currently recruiting mosques, households and participants to the study. Recruitment is expected to be complete by the end of October 2018.

## Additional file

Additional file 1: The SPIRIT 2013 Checklist: MCLASS II trial protocol*. (DOC 121 kb)

## Abbreviations

CACE: Complier average causal effect
CEAC: Cost-effectiveness acceptability curve
cRCT: Cluster randomised controlled trial
DALYs: Disability-adjusted life years
EQ-5D-Y: EuroQol- 5 Dimension Youth
EQ-5D: EuroQol- 5 Dimension
FI: Field investigators
IAQ: Indoor Air Quality
ICC: Intracluster correlation coefficient
ICERs: Incremental cost-effectiveness ratios
ID: Identifier
IF: Islamic Foundation
ITT: Intention-to-treat
LMICs: Low- and middle-income countries
M4bH: Muslims for better Health
MCLASS: Muslim Communities Learning About Second-hand Smoke
MRC: Medical Research Council
PedsQoL: Pediatric Quality of Life Inventory^TM^
PM_2.5_: Particulate matter less than 2.5 μm in diameter
SD: Standard deviation
SFH: Smoke-Free Homes
SGRQ: St George’s Respiratory Questionnaire
SHS: Second-hand smoke
TSC: Trial Steering Committee
UNION: The International Union against Lung Disease and Tuberculosis
WHO: World Health Organisation
